# Hypopharyngeal Reconstruction: Possibilities, Outcomes, and Updates for Improving the Human Health for Quality of Life

**DOI:** 10.1155/2022/6132481

**Published:** 2022-02-08

**Authors:** Hani Marzouki, Majda A. Addas, Mohammed Nujoom, Faisal Zawawi, Hatim Z. Almarzouki, Mazin Merdad

**Affiliations:** ^1^Department of Otolaryngology, King Abdulaziz University Hospital, Jeddah 21589, Saudi Arabia; ^2^Faculty of Medicine, King Abdulaziz University Hospital, Jeddah 21589, Saudi Arabia; ^3^Department of Radiology, Faculty of Medicine, King Abdulaziz University Hospital, Jeddah 21589, Saudi Arabia

## Abstract

Hypopharyngeal carcinoma is usually present at late stages, necessitating an aggressive line of management consisting of surgical procedures, chemotherapy, and radiation therapy, depending on the case. Practitioners tend to support total laryngectomies or total esophagostomies for most cases of hypopharyngeal carcinoma. The extensive procedures needed will most probably require, depending on the residual defect, a follow-up reconstructive procedure that might require utilizing flaps. Types of reconstructive methods and types of grafts or flaps used could be divided into a multitude of categories depending on the magnitude, shape, extension, and whether the underlying defect that is being reconstructed is circumferential or not. These reconstructive procedures are aimed at improving the quality of life, improving the aesthetic outcome, and restoring the functionality of the pharyngoesophageal segment. When it comes to hypopharyngeal cancer, the most common kind is squamous cell carcinoma (SCC), which has the worst prognosis of all the head and neck malignancies. Overall, the 5-year survival rate remains low, despite recent advancements in diagnostic imaging, radiation, and chemotherapy, as well as enhanced surgical methods and techniques. Hypopharyngeal malignancies are more probable than other tumors to present with advanced primary illness, with nodal metastasis a distinct possibility. The size and amount of local dissemination of the original carcinoma, as well as the extent of involvement of regional lymph nodes, are the most critical factors in predicting prognosis. Hypopharyngeal cancers are more likely than other head and neck cancers to manifest with distant metastases at the time of diagnosis. The appearance of second primary tumors, as well as the development of distant metastases, is a contributing factor to poor survival rate. Imaging techniques such as computed tomography (CT) and magnetic resonance imaging (MRI) with contrast remain the gold standard for evaluating hypopharyngeal carcinoma in the early stages. In most cases, imaging leads to an increase in the tumor stage at the time of presentation. *Objectives*. The main objectives are to review the research published about flaps, outline the optimum situations that will dictate the usage of a few of the most often used flaps for the rebuilding of the hypopharyngeal segment defects, and outline some of the complications associated with reconstruction. *Methods*. The processing was carried out with the title-specific search of the PubMed database using the query terms “hypopharyngeal carcinoma” and “reconstruction” to identify the most relevant articles without restricting publication dates. Information about the types of defects and methods of reconstruction was extracted from the reviewed articles. Two books were also reviewed, which were Regional and Free Flaps for Head and Neck Reconstruction (second edition) and Head and Neck Reconstruction: A Defect-Oriented Approach. *Conclusion*. Deciding the appropriate approach to a case should be individualized and should depend on the capabilities of the center, the defect's size and status, and lastly, the surgeon's training. The use of interpretation in the diagnosis of flaps can offer the best results in restoring functionality and vascularity and might also offer improved cosmesis.

## 1. Introduction

Globally, oral cancer is the sixth most common malignancy, according to the World Health Organization, and the most treatable. This category includes a collection of neoplasms that may affect any area of the mouth cavity, pharyngeal regions, or salivary glands [1]. The mouth cavity, pharyngeal regions, and salivary glands are all included in this group. Oral squamous cell carcinoma (OSCC) is the most frequent of all oral neoplasms and accounts for the vast majority of occurrences; nonetheless, this term is occasionally used interchangeably with it. According to current estimates, OSCC will account for more than 90 percent of all oral neoplasms worldwide [2]. The use of tobacco products such as cigarettes or betel quid, as well as the use of alcoholic beverages on a regular basis, is the most important risk factor for oral squamous cell carcinoma. Oral squamous cell carcinoma has been linked to both a low consumption of fresh fruits and vegetables and infection with high-risk human papillomavirus (HPV) genotypes in recent years [1, 3]. When it comes to oral squamous cell carcinoma, the Indian subcontinent is home to the highest incidence and prevalence rates, with the risk of acquiring oral squamous cell carcinoma being increased by the very prevalent practice of chewing tobacco, betel quid, and areca nut.

Cancers of the upper aerodigestive tract, also known as head and neck cancers, are mainly squamous cell carcinomas, which account for around 90 percent of all cases, with varied degrees of differentiation and a male to female predominance ratio of 10 : 1. Head and neck squamous cell carcinomas (HNSCC) are the sixth most common cancer in the world, accounting for around 4% of all malignancies in the United States and 5% in the United Kingdom [4]. Hypopharyngeal carcinomas often emerge at a late stage (stage 3 or stage 4), making cure less likely than in other cancers [5]. There are many critical prognostic markers for carcinoma of the hypopharynx, including the architecture and location of the tumor. Cigarette smoking is one of the most significant predisposing risk factors associated with the development of HNSCC [6]. Other potentially harmful variables include the intake of alcoholic beverages and infection with either high-risk human papillomavirus (HPV), particularly type 16, or the Ebstein–Barr virus (EBV) [7]. According to a carefully conducted study, 61 percent of oropharyngeal squamous cell carcinomas (OSCC) were HPV 16 positive, indicating that HPV infection is a significant predisposing risk factor for developing OSCC [8, 9]. A more favorable response to therapy was seen in patients with HPV-associated OSCC as well as a higher overall survival rate. Smoking, on the other hand, is a stronger predisposing factor that tends to make rehabilitation more difficult.

Research has shown a worse prognosis in patients with HPV-related tumors and a smoking history; ten packs-year was the cut-off used in a recent important study than in patients suffering from only an HPV-related OSCC [10, 11]. Patients falling with HPV implicated OSCC appear to be younger, have few comorbidities, and their OSCC tends to be more chemo- and radiosensitive [12]. Considered a less common form of head and neck malignancy, squamous cell carcinoma of the hypopharynx is considered that less than 3%–5% are head and neck carcinomas [8]. Squamous cell carcinoma of the hypopharynx exemplifies a carcinoma with a poor prognosis, high rates of multicentricity, submucosal spread, and regional and distal metastasis [13]. About 22.6% of patients in a retrospective series who were treated for hypopharyngeal squamous cell carcinoma experienced the presence of residual disease, which was up to 49.8% and had disease degenerated [8]. The incidences of upper aerodigestive tract cancers in the Kingdom of Saudi Arabia (KSA) in 2018, according to the World Health Organization (WHO), are shown in Table 1 [14]:

Early stages of head and neck cancer can be readily cured with surgery or radiotherapy with promising results; however, certain risk factors can be implicated in disease recurrence. In the era of organ preservation, practitioners are leaning more towards concomitant use of radiotherapy and chemotherapy instead of directly opting for surgery, especially when it comes to preserving the larynx [15, 16]. A significant improvement in results was noted when treated with chemotherapy and radiotherapy coincided, which was demonstrated in a study that reported a 67% complete remission rate using dual therapy [17]. Reconstruction poses a challenge due to the complex nature of the procedures. Performing a reconstructive procedure requires an experienced multidisciplinary team that should include a head and neck surgeon, radiation oncologist and medical oncologist, radiologist, dietician, dentist, pain physician, and swallowing physician. Advanced carcinomas of the hypopharynx or cervical esophagus usually have a poor vaticination and low long-term survivability; therefore, the surgery is considered a palliative surgery performed in a salvage setting and is preferred to be accomplished as a one-stage procedure rather than a delayed or multistage procedure. The goals of pharyngoesophageal reconstruction are not constrained to limiting the potential life-threatening postoperative complications, including protecting the great vessels, separation of respiratory and digestive tracts, and prevention of mediastinal infection, but also include improving the quality of life. Improving the quality of life is accomplished by the restoration of pharyngoesophageal function, especially the ability to produce speech, usually through speech rehabilitation, and reconditioning of the progression of the alimentary tract to allow the patient to resume an oral diet [18]. To achieve this, oftentimes, flaps or grafts are used as a part of the reconstruction. In general, the types of reconstructive methods and types of grafts or flaps used could be divided into many categories depending on the magnitude, shape, extension, and whether the underlying defect that is being reconstructed is circumferential or not. The defect could be repaired by primary mucosal repair, local skin flap, regional flaps that include the pectoralis major myocutaneous flap, deltopectoral flap, trapezius flap, and latissimus dorsi flap. Other methods include enteric flap transposition, which consists of but is not limited to gastric pull-up and colon transposition. Microvascular enteric free flaps involving jejunal free flaps and gastro-omental flaps, and microvascular fasciocutaneous free flaps which encompass the radial forearm free flap and the anterolateral thigh free flap may also be used.

## 2. Classification of Defects of the Pharyngoesophageal Segment

Again, the goals of reconstructive procedures that are concerned with improving the quality of life are breathing without a tracheostomy, speaking with an artificial valve, and swallowing without aspiration. The ability to restore the pharyngoesophageal segment's functionality depends on the extent of preservation of the larynx after the initial surgery and whether or not separate conduits for breathing and swallowing have been established. Disa et al. described one method of classification of defects of the pharyngoesophageal segment following total laryngectomy [19]:

Stage 1 is normal, which means that cancer has not spread to lymph nodes or other regions of the body (Tis, N0, and M0).

During stage I, the tumor is no more than 2 cm in diameter, and cancer has not spread to lymph nodes or other regions of the body (T1, N0, and M0).

During stage II, the tumor is between 2 and 4 cm in size, but cancer has not progressed to the lymph nodes or other regions of the body.

During Stage III: (T2, N0, M0), one or both of the following conditions are met: even if the tumor is greater than 4 centimeters in diameter or has reached the epiglottis, the malignancy has not migrated to lymph nodes or other regions of the body at this time (T3, N0, M0).

With the exception of the epiglottis, the tumor has not spread to surrounding tissues. Cancer has been found in one lymph node on the same side as the initial tumor, and it is 3 cm or less in size, with no evidence of endocrine disruption (ENE) (T1–T3, N1, and M0). Cancer has not migrated to any other sections of the body (T1–T3).

Stage IVA is characterized by the presence of one or more of the following conditions:

It is possible that the tumor has reached the larynx, the tongue, the jawbone, the roof of the mouth, or the jawbone has been invaded by the tumor. Despite the fact that cancer has progressed to a single lymph node, it has not moved to other regions of the body (T4a, N0 or N1, and M0).

Technically, there is not much of a difference in the approach to reconstructing a *type I* and a *type II* defect as long as each of these defects has a useable, well-vascularized pharyngeal strip that spans the entire volume of the defect. This allows closure of the defect without having to resort to a “patch” for closure.

**Figure 1:**
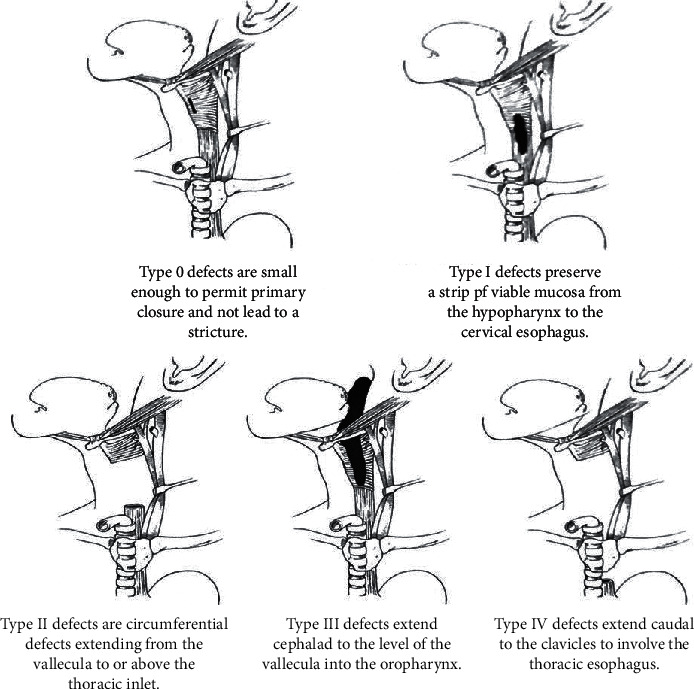
Pharyngoesophageal defect classification scheme [20].

Figure 1 represents the pharyngoesophageal defect classification scheme of various human bodies.

### 2.1. Primary Mucosal Repair

Primary mucosal repair is usually used in partial pharyngeal defects, which are typically associated with type 0 defects. It is mostly associated with desirable speech outcomes but is sometimes associated with stricture formation if the remnant's length is insufficient [21]. Hui et al. [22] reported the absence of dysphagia after total laryngectomy in 42 patients in a study consisting of 52 patients where the lengths of the pharyngeal remnant were 1.5 cm relaxed and 2.5 cm stretched, which led to the conclusion that a remnant's length should fall within the 1.5 cm to 2.5 cm range to be eligible for primary mucosal repair with preservation of the swallowing function. More extensive defects are reasonably constructed using a pedicled flap, generally a pectoralis major flap. Free flaps, such as the radial forearm flap and the anterolateral thigh flap, may be utilized. These flaps are known as “patch” grafts.

### 2.2. Total Defects in Pharyngolaryngectomy

If the desired bottom anastomosis lies over the clavicle after total circumferential pharyngolaryngectomy, multiple reconstruction options exist. These options include the jejunal free flap (JFF), tubed radial forearm free flap (RFFF), gastro-omental free flap (GOFF), and a tubed anterolateral thigh free flap (ALT)[23].

### 2.3. Lower Anastomosis under the Clavicles

If the resection extends under the clavicles, a gastric pull-through or colonic transposition flap may be utilized [24]. Both these procedures carry increased morbidity and mortality because of the requirement to enter multiple visceral cavities. When gastric pull-up or colonic transposition is utilized, care must be taken regarding reflux, regurgitation, persistence of peristaltic movement, and dysphagia [25].

### 2.4. Regional Flaps

#### 2.4.1. Pectoralis Major Myocutaneous Flap

Due to its large surface area, the pectoralis major muscle is used in reconstruction after total laryngectomy, repair of circumferential pharyngoesophageal defects, noncircumferential type I defects, or defects in areas with impaired vascularity and healing due to previous therapy. It is also used as a medially based flap to provide protection for great vessels and obliteration of dead space following mediastinal dissection for recurrent laryngeal cancer after total laryngectomy [26]. It facilitates reconstruction as a single-stage procedure of circumferential pharyngoesophageal defects, and it is considered easy to harvest when the patient is in the spineless position. The big bulk, the thickness of the pectoralis major muscle, skin paddle, and subcutaneous fat makes the flap too thick to be effectively tubed in cases of reconstruction of circumferential defects in most patients. When healing by secondary intention occurs, the potential for wound contraction significantly increases the chance of stricture formation. Modifications can be made to the flap to make it more useable, but they pose a new set of complications, including flap necrosis. Relatively thin males are the best candidates when using this flap due to the smaller bulk and less subcutaneous fat [27]. The vascular anatomy of the pectoralis major flap is found on the thoracoacromial artery.

The reconstruction results, using the pectoralis major myocutaneous flap, are more favorable when it is applied to reconstruct noncircumferential defects. Among 24 cases reported in the literature, swallowing was achieved in most patients after an interval of 7 to 14 days, and the incidence of fistula formation was 13% [28–30]. Schecter [31] reported poor nasoesophageal speech among 27 patients who underwent pectoralis myocutaneous flap pharyngoesophageal reconstruction.

## 3. Deltopectoral Flap

The deltopectoral (Dp) flap or the Bakamjian flap is a fasciocutaneous flap used to reconstruct type I and type II defects of the pharyngoesophageal segment and facial tumors [32]. Because of the texture, color, and flexibility of the skin in this region, the Dp flap has a tendency to provide functionally and aesthetically pleasing results [33]. The Dp flap is usually utilized in patients who, due to comorbidities, cannot undergo more complex methods of reconstruction. The method of reconstruction used with this flap is usually staged with a 3- to 5-week delay period between the primary and secondary procedures to allow the harvest of a large surface area. This delay results in delayed swallowing and is crucial to minimize the probability of distal flap necrosis when a flap extending into the shoulder is raised. This flap's vascularity is more reliable than that of the cervical skin flaps due to the latter's arbitrary nature of vascularity [34, 35]. The blood flows from the parasternal perforators of the internal mammary artery and vein, which transverse the intercostal spaces. To facilitate the safe transfer of the flap in terms of vascularity when moving further away (laterally) from the internal mammary perforators (the principal perforators), Taylor et al. [36] developed the “angiosomes concept.” The concept showed that the blood flows from the main angiosome and adjacent angiosome is reliable, but the additional angiosome, such as that above the deltoid muscle, is at risk of experiencing ischemic necrosis. Once a Dp flap is expanded lateral to the deltopectoral groove, its reliability is decreased. Some of the unfavorable postoperative complications that have been reported in the literature are flap necrosis, fistula formation, and stenosis, with a percentage of 67% among 12 patients of tubed deltopectoral flap pharyngoesophageal reconstruction [37]. Regaining the ability to swallow took about ten weeks to achieve and holds an average percentage of 83%, but it should be considered that the patients underwent an average of 3.8 methods to complete the construction. Only some patients were able to attain fluent neoesophageal speech next to the reconstruction of the hypopharynx using this flap [31].

### 3.1. Free Flaps

#### 3.1.1. Radial Forearm Flap

The radial forearm flap (RFF), also called the Chinese flap, is a fasciocutaneous flap that was first mentioned by Yang et al. [38] in 1981. The skin of the radial forearm is thin and flexible; therefore, it can be tubed and shaped easily according to the shape of the defect. It is also highly vascularized, which makes it very reliable in a sense where flap ischemia and necrosis are not the main complications. They are used to restore noncircumferential type I defects when primary mucosal repair cannot be achieved, circumferential type II when the thoracic esophagus is intact, and intraoral reconstruction following ablative surgery, where it has been used in almost every portion of the oral cavity [39–41], where it helped in the accommodation of a dental prothesis afterward. It is also the flap of choice for patients with an intact larynx in whom a portion of the wall of the hypopharynx is removed. “Through-and-through” imperfection of the mucosa and skin can also be reconstructed by applying the radial forearm flap where the skin paddle is used to reconstruct the pharyngoesophagus and the outer neck skin can be covered with a skin graft otherwise a pectoralis major flap with a skin island. At the plane of the antebrachial fossa, the brachial artery bifurcates, giving rise to the radial and ulnar arteries that contribute to the lower arm and the hand. The radial artery yields the deep palmar arch, while the ulnar artery terminates in the superficial palmar arch. Because of this, harvesting a radial forearm flap will dictate total reliance on the ulnar system to retain the vascularity of the hand, knowing that the radial artery will be completely disrupted during the harvest [42]. A traditional Allen's test is executed to determine the safety of sacrificing the radial artery, resulting in ischemia of the hand.

The forearm skin is supplied by four arterial systems through a multitude of subcutaneous and musculocutaneous perforators. These four vessels are the radial, ulnar, anterior, and posterior interosseous arteries. The venous drainage system is fractionated into deep and superficial systems. The superficial system is present in the subcutaneous tissue of the top limb and consists of the cephalic and basilic veins. The cephalic vein runs on the anterio-lateral aspect of the top limb, empties into the axillary vein, and is connected to the basilic vein at the elbow by the median cubital vein. The RFF is also sometimes described to be an osteo fasciocutanous flap, and the dimensions of the bone that can be safely harvested are limited by the need to ensure the structural integrity of the remaining radial segment. The skin of the forearm derives its sensory nerve supply from the anterior and posterior branches of the lateral cutaneous nerve of the forearm, a carrying on of the musculocutaneous nerve, and the anterior and posterior branches of the medial cutaneous nerve of the forearm. The muscles of the anterior fascial compartment of the forearm are contributed by the median nerve and its branches, except for the flexor carpi ulnaris and the medial part of the flexor digitorum profundus, which are supplied by the ulnar nerve. The muscles of the posterior fascial compartment of the forearm are contributed by the radial nerve [43]. Sensory loss following the disruption of the superficial branches of the radial nerve and sampling of the antebrachial cutaneous nerve has been reported, while donor site infection and loss of function are not major consequences of using this flap unless the flap being used is an osteocutaneous flap.

## 4. Result Analysis: Case Presentation

**Figure 2:**
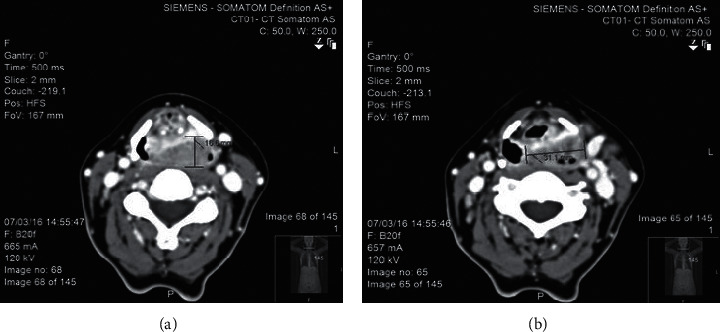
Prechemotherapy and radiotherapy CT scans. (a) and (b) show a left parpharyngeal mass measuring 31.1 mm in width and 16.6 mm in height compressing the airway from the ipsilateral side.

**Figure 3:**
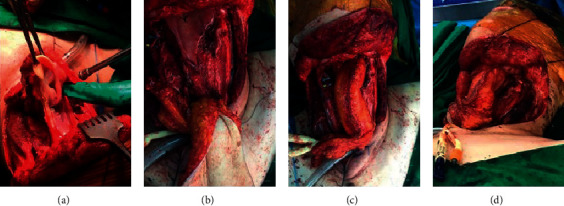
Total laryngectomy and reconstruction using a tubed radial forearm flap. (a) The larynx being removed with the primary tumor. (b) Defect is shown with flap in the setting. (c, d) Radial forearm free flap post.

**Figure 4:**
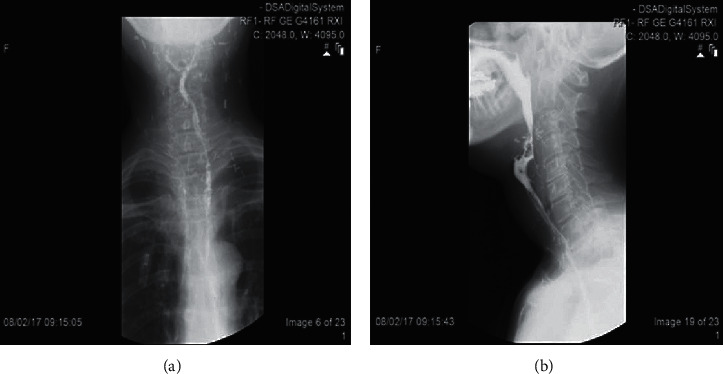
Barium swallow study. (a, b) Gastrografin swallow study done on the seventh postoperative day showing the contrast going smoothly with no signs of obstruction or fistulae.

A 54-year-old female exsmoker was put forward to our hospital complaining of progressive dysphagia and pain for the duration of one month. Examination showed a left-sided neck mass at level II measuring 2 cm × 2 cm in size. The fibro-optic scope showed a mass in the left pyriform sinus and postcricoid fossa with fixation to the left vocal fold. The patient was sent for chemotherapy and radiotherapy. A CT scan was done earlier than the chemotherapy and radiotherapy. The CT scan (Figures 2 and 3) showed a hypopharyngeal mass, left pyriform sinus, and postcricoid fossa mass. Panendoscopy with biopsy was performed. The biopsy showed invasive, moderately differentiated squamous cell carcinoma. The patient has gone through the total laryngopharyngectomy, which left a defect that was reconstructed using a tubed radial forearm flap.  Figure 4 shows a series of pictures taken intraoperatively, and show a barium swallow study performed postoperatively. The patient stayed at the hospital for 10 days postoperatively where she was NPO at first. On day seven postoperatively, a barium swallow was performed which showed no leak and the diet progressed gradually. The patient then was discharged home and has been following up at the outpatient clinic for four years now. There was no recurrence or major complications. The voice button was used through the transesophageal voice prosthesis (TEP) for speech restoration. The patient had one episode of dysphagia during her second postoperative year. The dysphagia was corrected after undergoing esophageal dilation. Now, in the fourth year postoperatively, the patient is still doing well and is following up at the head and neck clinic for annual surveillance.

Figure 4 gives the swallow study of the neck region of various cross-sectional views.

### 4.1. Anterolateral Thigh Free Flap

The anterolateral thigh (ALT) free flap provides a flap with a great harvest surface area, reduced donor site morbidity, and potentially reduced stricture formation when compared to other types of flaps, especially radial forearm flaps. Anterolateral thigh free flaps are utilized for an abundance of head and neck defects that need to be rebuilt. ALT is the flap of choice in thin patients for the rebuilding of noncircumferential or circumferential types I, II, and III pharyngoesophageal defects. It is also used to reconstruct the oral cavity after resection of tumors resulting in defects of the tongue, buccal mucosa, palate, and lips [44–47]. In cases of “through-and-through” pharyngoesophageal defects, this flap can be greatly utilized in reconstruction. It can also be used for reconstruction of the pharyngoesophageal segment and rebuilding of soft tissue imperfection of the skull base, midface, and scalp. These flaps are different in thickness depending on body habitus, gender, ethnicity, flap design, and harvest techniques. Flap thinning procedures can be done to reduce the volume of subcutaneous fat when thinner flaps are needed. A certain amount of subcutaneous tissue needs to be transferred with the ALT-free flap. Usually, when a flap is harvested, surgeons will try to close the donor site. The possibility of achieving primary wound closure is based on the width of the harvested flap, taking into consideration the fact that the maximum width that can be harvested while still allowing primary closure to take place is between 8 cm and 10 cm, even though a 12 cm defect has been reported in the literature [44]. The sensory supply of the anterolateral thigh is supplied by the lateral cutaneous femoral nerve, the superior perforator nerve, and the median perforator nerve. The lateral femoral cutaneous nerve, which is located along a line in the middle of the anterior superior iliac spine and the patella, can be used to reinnervate the skin paddle of the ALT flap. When discussing the outcomes after reconstruction with the ALT flap, a study reported that all of its patients regained oral intake with an average of 5.8 weeks postoperatively. No life-threatening complications such as carotid blowout, sepsis, deep space infections, or abscesses were reported at the donor site. Late complications were reported in 6 patients, which included stricture and fistula in two patients, stricture alone in one patient, fistula alone in two patients, and a lymphocele in one patient [48].

### 4.2. Gastric Pull-Up

**Figure 5:**
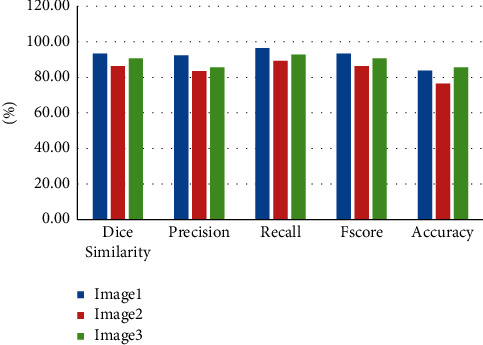
Performance metrics.

Just like hypopharyngeal carcinomas, carcinoma of the cervical esophagus is an unfamiliar yet very important matter, and the surgical approach is sometimes as aggressive as the carcinoma itself. Gastric pull-up is a type of gastric transposition which is indicated to reconstruct type IV hypopharyngeal defects in patients who have undergone therapy for squamous cell carcinoma with the involvement of the cervical and thoracic esophagus [49]. The gastric pull-up method of reconstruction is preferred in cases where a transcervical approach to enteric anastomosis seems feasible and poses a great risk of morbidity and mortality where it facilitates reinstating the progression with vascularized tissue without the need for vascular anastomosis. It is usually done in a previously irritated setting, with patients being weak and malnourished. The blood flow is supplied by the right gastric artery and its gastroepiploic arcade, which should be carefully handled during the pull-up to avoid necrosis. There are a multitude of contraindications and complications regarding gastric-pull-up. Previous major abdominal surgery, cardiac or pulmonary comorbidities, esophageal varices, and portal hypertension are all contraindications. This technique of reconstruction poses a high risk of morbidity and mortality; therefore, careful selection of appropriate patients is very crucial for survival. The gastric pull-up flap cannot be stretched to extend more cephalad to reach the oropharynx, which might limit its usage. In an attempt to minimalize postoperative complications, thoracoscopic and laparoscopic approaches are favored, but with the caveat of the need for an experienced team. Previous radiation therapy and gravitational pull on the area might predispose to delayed wound healing. Some of the complications of gastric-pull are mediastinitis and fistula formation. A study of twenty-four patients reported postreconstruction complications in 13 patients. These complications included anastomotic fistulas in 9 patients, subphrenic abscess in one patient, and lymphorrhea in one patient. Regarding swallowing, 14 patients were able to regain oral intake, three had mixed feeding, and seven patients did not achieve normal feeding [50].  Table 2 represents the performance metric, and Figure 5 represents the graphical representation.

Figure 5 represents the performance metrics of the proposed work.

## 5. Conclusion

When having surgery for hypopharyngeal cancer, the morbid consequences of the procedure are compounded by the difficulty of rebuilding the resultant defect postoperatively. The degree of the flaw, whether the defect is circumferential or not, and the state in which the fault is found all influence how well the defect may be repaired or rebuilt. Minor or type 0 defects may be repaired by primary mucosal healing or by the use of an autologous skin transplant if the defect is small or of a type 0 nature.

Flaps are used to repair more significant flaws on a more frequent basis. Pectoralis major myocutaenous flaps are used for reconstruction following complete laryngectomy, repair of circumferential pharyngoesophageal defects, correction of noncircumferential type I pharyngoesophageal defects, and repair of defects in regions with poor vascularity and healing due to prior treatment. A stricture may occur in the tubing of the pectoralis major myocutaneous flap, which is why it is not recommended for use in this situation. The deltopectoral flap is a fasciocutaneous flap that is used to restore type I and type II defects in the pharyngoesophageal segment, as well as facial tumors in the pharyngoesophageal segment. The fact that it is malleable allows for simple contouring and a fair color match with respect to the neck skin, which is an important cosmetic benefit. Due to the sensitivity of its vascular nature and the possibility of flap necrosis, caution should be used while harvesting and insetting this flap throughout the procedure. When employing this flap, the consequence of your speech is not favorable. In order to correct the problem, the stomach is raised to the level of the defect, which has serious repercussions. There are several difficulties associated with gastric pull-up, as well as high rates of morbidity and death. Gastric reflux, fistulas, mediastinitis, dysphagia, and dysphonia are just a few of the consequences associated with the condition. Long-term survival after gastric pull-up is not very high, but it may be used to improve the quality of life in patients who have had satisfactory postoperative swallowing outcomes following gastric pull-up. The technique of treatment and the method of reconstruction should be customized to meet the specific demands of each individual patient. The protocol for the treatment of hypopharyngeal cancer, as well as the kind of construction that is used, is defined by the capabilities of the center, the size and condition of the defect, and, lastly, the training of the surgeon doing the procedure. Flaps have the potential to provide the finest outcomes in terms of restoring functioning and vascularity, as well as better cosmesis [5].

## Figures and Tables

**Table 1:** Different analysis of cancer.

Type of cancer	New cases (%)	Rank by incidence worldwide	Percentage of death (%)	5-year survival prevalence
Nasopharynx	1.7	18th	2.1	4.12
Lip and oral cavity	1.5	19th	1.6	3.27
Oesophagus	1.1	22nd	2.2	0.81
Larynx	0.63	25th	0.86	1.38
Hypopharynx	0.21	29th	0.22	0.32
Oropharynx	0.07	32nd	0.15	0.19

**Table 2:** Performance metrics.

	Image1	Image2	Image3
Dice similarity	93.36%	86.3%	90.66%
Precision	92.34%	83.46%	85.65%
Recall	96.48%	89.33%	92.76%
*F*-score	93.36%	86.3%	90.66%
Accuracy	83.76%	76.56%	85.63%

## Data Availability

The data that support the findings of this study are available on request from the corresponding author.
